# Microwave-Assisted Synthesis of Arene Ru(II) Complexes Induce Tumor Cell Apoptosis Through Selectively Binding and Stabilizing *bcl*-2 G-Quadruplex DNA

**DOI:** 10.3390/ma9050386

**Published:** 2016-05-17

**Authors:** Yanhua Chen, Qiong Wu, Xicheng Wang, Qiang Xie, Yunyun Tang, Yutao Lan, Shuangyan Zhang, Wenjie Mei

**Affiliations:** 1 School of Pharmacy, Guangdong Pharmaceutical University, Guangzhou 510006, China; chenyinwah423@163.com (Y.C.); 13533260720@163.com (Q.W.); 13724085853@163.com (S.Z.); 2 The First Affiliated Hospital of Guangdong Pharmaceutical University, Guangzhou 510080, China; 13902400598@126.com (X.W.); 15920134017@163.com (Y.T.); 3 The Third Affiliated Hospital of Sun Yat-Sen University, Guangzhou 440100, China; doctorxie@126.com; 4 School of Nursing, Guangdong Pharmaceutical University, Guangzhou 510310, China

**Keywords:** arene Ru(II) complexes, microwave-assisted synthesis, apoptosis inducer, selective stabilizing, *bcl-2* G-quadruplex DNA

## Abstract

A series of arene Ru(II) complexes coordinated with phenanthroimidazole derivatives, [(η^6^-C_6_H_6_)Ru(l)Cl]Cl(**1b** L = *p*-ClPIP = 2-(4-Chlorophenyl)imidazole[4,5f] 1,10-phenanthroline; **2b** L = *m*-ClPIP = 2-(3-Chlorophenyl)imidazole[4,5f] 1,10-phenanthroline; **3b** L = *p*-NPIP = 2-(4-Nitrophenyl)imidazole[4,5f] 1,10-phenanthroline; **4b** L = *m*-NPIP = 2-(3-Nitrophenyl) imidazole [4,5f] 1,10-phenanthroline) were synthesized in yields of 89.9%–92.7% under conditions of microwave irradiation heating for 30 min to liberate four arene Ru(II) complexes (**1b**, **2b**, **3b**, **4b**). The anti-tumor activity of **1b** against various tumor cells was evaluated by MTT assay. The results indicated that this complex blocked the growth of human lung adenocarcinoma A549 cells with an IC_50_ of 16.59 μM. Flow cytometric analysis showed that apoptosis of A549 cells was observed following treatment with **1b**. Furthermore, the *in vitro* DNA-binding behaviors that were confirmed by spectroscopy indicated that **1b** could selectively bind and stabilize *bcl-2* G-quadruplex DNA to induce apoptosis of A549 cells. Therefore, the synthesized **1b** has impressive *bcl-2* G-quadruplex DNA-binding and stabilizing activities with potential applications in cancer chemotherapy.

## 1. Introduction

Arene Ru(II) complexes have long been considered one of the most promising candidates for anti-cancer drug therapy due in part to their low toxicity and remarkable anti-cancer activity [1]. Numerous arene Ru(II) complexes have been designed, and their anti-tumor activity and DNA binding behavior, as well as their underlying mechanisms of action, have been extensively investigated. Consequently, it was shown that arene Ru(II) complexes can bind and disturb the replication of DNA, and thus induce apoptosis and tumor cell cycle arrest [2,3,4,5,6,7,8]. Arene Ru(II) complexes coordinated by phenanthroline ligands have the potential to prevent tumor growth at low initial concentration by intercalating the base-pairs of duplex DNA [9]. RAPTA-B ([Ru(η^6^-C_6_H_6_)(pta)-Cl_2_]) and RAPTA-C ([Ru(η^6^-p-C_6_H_4_MeiPr)(pta)Cl_2_]) has the potential to block tumor metastasis in a CBA mouse model of human breast cancer by a mechanism that is partly dependent on inhibiting angiogenesis [10]. In addition, the arene Ru(II) complex RM175 ([(η^6^-biphenyl)Ru(ethylenediamine)-Cl]^+^) has the capacity to suppress tumor metastasis *in vivo* and block migration and invasion by enhancing cell–cell re-adhesion and dampening the release of matrix metalloproteinases (MMPs) [11]. In recent times, it has been determined that arene Ru(II) complexes coordinated with phenanthroimidazole derivatives like [(η^6^-C_6_H_6_)Ru(H_2_iip)Cl]Cl (RAWQ11) could effectively inhibit the migration and invasion of breast cancer cells. Further studies have shown that RAWQ11 induces DNA damage that is mediated by S-phase arrest, activates the Protein Kinase B (AKT) signaling pathway, and thus inhibits the formation of invadopodia [12,13,14]. Collectively, these studies provide encouraging support that arene ruthenium (II) complexes could be used in the clinic to combat tumor invasion and migration. In general, arene Ru(II) complexes can be obtained by refluxing the precursors [{RuCl (μ-Cl)(η^6^-C_6_H_6_)}_2_] with the corresponding ligand; however, this method is limited due to the low turnover and difficulties in purifying the obtained products [15,16]. In 1986, Giguere first reported the application of commercial microwave ovens in organic synthesis [17]. Subsequently, microwave-assisted synthesis technology has been extensively developed in the fields of chemical synthesis, materials science, and biotechnology due to properties of both high efficiency and high yield [18,19,20]. Metal organic small molecules are generally obtained at room temperature by stirring for a couple hours. In recent years, use of microwave-assisted synthesis technology in the preparation of metal complexes [21,22], especially ruthenium complexes [23,24], could significantly increase the yield of many complexes to about 90% in a shorter period of time (<30 min). For example, in 2006, the first use of microwave-assisted technology in the synthesis of ruthenium complexes, which could not be synthesized by a more conventional approach, was obtained under microwave radiation [25]. In 2007, a new ruthenium complex which cannot be synthesized by a general method was obtained under microwave radiation [26]. However, to date, comparatively few reports are available on the application of microwave-assisted technology to prepare arene Ru(II) complexes.

**Scheme 1:**
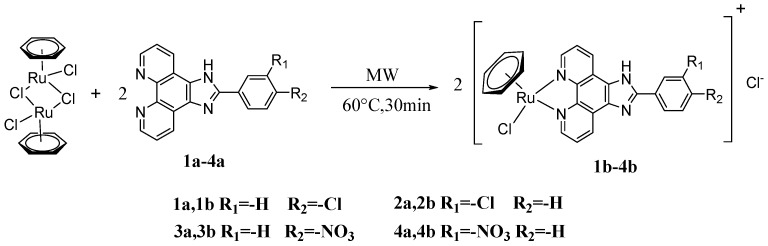
Microwave-assisted synthesis route for arene Ru(II) complexes.

Over-expression of the *Bcl-2* proto-oncogene disrupts the therapeutic action of current cancer treatment regimes by inducing apoptosis of tumor cells [27]. The broad expression of *Bcl-2* in a variety of tumors, together with its function in resisting chemotherapy-induced apoptosis, makes *bcl-2* a rational target for anti-cancer therapy. The human *bcl-2* gene contains two promoters, referred to as P1 and P2 [28]. The P1 promoter, which is a GC-rich region that is capable of forming G-quadruplexes, is involved in the regulation of *bcl-2* gene expression, and thus modulation of the appearance of cancer [29]. When one rationally designs drugs that target the *bcl-2* G-quadruplex DNA for cancer chemotherapy, increased selectivity for G-quadruplex DNA and low cytotoxicity are key factors to consider in this process [30]. There is clear evidence that only a few Ru(II) complexes promote the formation and stabilization of G-quadraplex DNA [31]. Thomas and co-workers studied the binding behavior of a dinuclear Ru(II) complex with different quadruplex DNA structures [32]. It was found the Ru-complexes displayed sequence selectivity and high-affinity binding to duplex DNA through groove binding [33]. Our research group has shown that arene Ru(II) complexes coordinated with phenanthroimidazole exhibit great anti-tumor activity and low toxicity to normal cells, owing to their ability to bind and stabilize the G-quadruplex structure of *c-myc* and subsequent blocking of the replication of *c-myc* oligomer [24]. This finding might suggest that arene Ru(II) complexes show stronger binding affinity to *bcl-2* G-quadruplex DNA than to duplex DNA (calf thymus DNA). In the present study, a series of arene Ru(II) complexes (Scheme 1) were synthesized under microwave irradiation. The molecular mechanisms through which the arene Ru(II) complexes caused cancer cell death were also elucidated, and the results indicated that arene Ru(II) complexes inhibited cell proliferation by inducing tumor cells apoptosis. Thus, a number of approaches were used to establish whether the induced tumor cell apoptosis by synthesized arene Ru(II) complexes was caused by an interaction between the complexes and DNA. The DNA-binding properties of complex **1b** with calf thymus deoxyribonucleic acid (CT-DNA) and *bcl-2* G-quadruplex DNA was respectively studied in this report. The results indicated that arene Ru(II) complexes coordinated with phenanthroimidazole selectively bind and stabilize the *bcl-2* G-quadruplex DNA structure, leading to apoptosis of A549 cells.

## 2. Experimental Section

### 2.1. Chemicals

Ruthenium(III) chloride hydrate was obtained from Mitsuwa Chemicals (Tokyo, Japan). 1,10-Phenanthroline monohydrate, 1,3-cyclohexadiene, and 2-chlorobenzaldehyde were purchased from Sigma-Aldrich (St. Louis, MO, USA). All chemicals including solvents were obtained from commercial vendors and used as received. Calf thymus DNA (CT-DNA) was purchased from Guangzhou Ruizhen Biotechnology Co. (Guangzhou, China). *bcl-2* G-quadruplex DNA (5′-CGGGCGCGGGAGGAAGGGGGCGGGAGC-3′) were purchased from Sangon Biotech Co., Ltd. (Shanghai, China) and formed a G-quadruplex conformation by renaturation at 4 °C for 24 h after 90 °C denaturation for 5 min, as stipulated by methods in other studies [34]. 1,10-Phenanthro-line-5,6-dione was prepared by a similar method reported in the literature [35]. [{RuCl(μ-Cl)(η^6^-C_6_H_6_)}_2_] was synthesized according to literature [36]. All aqueous solutions were prepared with doubly distilled water. The Tris-HCl buffer was obtained from Tris (10 mM) and NaCl (100 mM), and the pH value was adjusted to 7.2 with HCl (0.1 mol); this buffer was used for ultraviolet (UV) titration, fluorescence quenching thermal denaturation, circular dichroism (CD) spectra of CT-DNA. The Tris-HCl buffer was obtained from Tris (10 mM) and KCl (100 mM), and the pH value was adjusted to 7.4 with HCl (0.1 mol); this buffer was used for ultraviolet (UV) titration, fluorescence quenching thermal denaturation, circular dichroism (CD) spectra of of G4-DNA.

### 2.2. Instruments

The arene Ru(II) complexes were synthesized using an Anton Paar GmbH monowave 300 microwave reactor (Anton Paar GmbH, Graz, Austria). The ^1^H NMR and ^13^C NMR spectra were recorded on a Bruker DRX2500 spectrometer. The Ultraviolet (UV) titration were recorded on a Shimadzu UV-2550 spectrophotometer, the steady-state emission spectra were recorded on a RF-5301 fluorescence spectrophotometer (Shimadzu Corporation, Kyoto, Japan), and the CD spectra were recorded on a Jasco J810 circular dichroism spectrophotometer (JASCO Corporation, Osaka, Japan).

### 2.3. Synthesis and Characterization

#### 2.3.1. Synthesis of **1a**, **2a**, **3a**, and **4a**

The ligand 1,10-phenanthroline-5,6-dione derivatives were prepared by a similar method according to previous literature we have reported, with some modifications [35]. A solution containing 1,10-phenanthroline-5,6-dione (1.6 mmol, 347 mg), substituted benzaldehyde derivatives (1.6 mmol), 20 mL of HAc and NH_4_Ac (33 mmol, 2.53 g), was heated at 110 °C under reflux for 4 h. Then, 20 mL of water was added and the pH value was adjusted to 7.0 at room temperature. The solution was filtered and dried in vacuum to obtain a yellow precipitate. The product was purified in a silica gel column by using ethanol as eluent. **1a**: yield 65.9%; **2a**: yield 70.5%; **3a**: yield 75.5%; **4a**: yield 72.5%.

#### 2.3.2. Microwave-Assisted Synthesis of Complex [(η^6^-C_6_H_6_)Ru(*p*-ClPIP)Cl]Cl **1b**

The arene Ru(II) complex **1b** were prepared according to literature, but with some modifications. A mixture of [{RuCl(μ-Cl)(η^6^-C_6_H_6_)}_2_] (0.10 mmol, 50.0 mg) and p-ClPIP **1a** (0.20 mmol, 66.0 mg) in dichloromethane (20 mL) was heated at 60 °C under microwave irradiation. A yellow precipitate was obtained after rotary evaporation. Then, it was purified by recrystallisation from distilled water, yields 99.0 mg (90.7%). ESI-MS (in MeOH): *m*/*z* 581.0, (Calculation(cal.)); *m*/*z* 546.1, ([M-Cl]^+^). ^1^H NMR (500 MHz, DMSO) δ: 14.73 (s, 1H), 10.08~9.69 (m, 2H), 9.36 (s, 1H), 8.39 (d, *J* = 8.6 Hz, 2H), 8.22 (s, 2H), 7.74 (d, *J* = 8.6 Hz, 2H), 6.33 (s, 6H). ^13^C NMR (126 MHz, DMSO-*d*_6_) δ 153.02 (s), 148.39 (s), 143.15 (s), 132.51 (s), 128.49 (s), 129.56 (s), 125.85 (s), 115.69 (s), 87.13 (s).

#### 2.3.3. Microwave-Assisted Synthesis of Complex [(η^6^-C_6_H_6_)Ru(*m*-ClPIP)Cl]Cl **2b**

**2b** was prepared using the method described above, but with *m*-ClPIP **2a** (0.20 mmol, 66.0 mg) instead of **1a**. A yellow precipitate was obtained, yields 101.0 mg (92.7%). ESI-MS (in MeOH): *m*/*z* 581.0, (cal.); *m*/*z* 546.1, ([M-Cl]^+^). ^1^H NMR (500 MHz, DMSO-*d*_6_) δ: 9.96 (dd, *J* = 5.3, 1.1 Hz, 2H), 9.35 (s, 2H), 8.43 (d, *J* = 11.5 Hz, 1H), 8.38 (dd, *J* = 10.3, 5.3 Hz, 1H), 8.20 (dd, *J* = 8.1, 5.4 Hz, 2H), 7.62 (m, 2H), 6.33 (s, 6H). ^13^C NMR (126 MHz, DMSO-*d*_6_) δ 155.31 (s), 150.50 (s), 144.68 (s), 135.21 (s), 133.81 (s), 132.45 (s), 129.61 (s), 127.35 (s), 126.46 (s), 88.06 (s).

#### 2.3.4. Microwave-Assisted Synthesis of Complex [(η^6^-C_6_H_6_)Ru(*p*-NPIP)Cl]Cl **3b**

**3b** was prepared using the method described above, but with *p*-NPIP **3a** (0.20 mmol, 68.2 mg) instead of **1a**. A yellow precipitate was obtained, yields 100.0 mg (89.9%). ESI-MS (in MeOH): *m*/*z* 592.4, (cal.); *m*/*z* 556.1, ([M-Cl]^+^). ^1^H NMR (500 MHz, DMSO) δ: 9.92 (m, 2H, phen-H), 9.20 (m, 2H, phen-H), 8.73 (m, 2H, phen-H), 8.44 (m, 2H, Ar-H), 8.10 (m, 2H, Ar-H), 6.32 (s, 6H). ^13^C NMR (126 MHz, DMSO-*d*_6_) δ 153.92 (s), 143.66 (s), 132.85 (s), 128.80 (s), 127.53 (s), 126.08 (s), 124.71 (s), 87.17 (s).

#### 2.3.5. Microwave-Assisted Synthesis of Complex [(η^6^-C_6_H_6_)Ru(*m*-NPIP)Cl]Cl **4b**

**4b** was prepared using the method described above, but with *m*-NPIP **4a** (0.20 mmol, 68.2 mg) instead of **1a**. A yellow precipitate was obtained, yields 102 g (91.7%). ESI-MS (in MeOH): *m*/*z* 592.4, (cal.); *m*/*z* 556.1, ([M-Cl]^+^). ^1^H NMR (500 MHz, DMSO) δ: 8.0 (m, 2H, phen-H), 7.7 (m, 2H, phen-H), 7.4 (m, 2H, phen-H), 6.33 (s, 6H). ^13^C NMR (126 MHz, DMSO-*d*_6_) δ 161.43 (s), 153.1 (s), 149.08 (s), 142.91 (s), 133.11(s), 131.97 (s), 128.80 (s), 115.04 (s), 113.03 (s), 87.03 (s).

### 2.4. Biochemical Mechanism

#### 2.4.1. Cell Lines and Cell Culture

Human cancer cell lines, human lung adenocarcinoma A549 cells, human hepatocarcinoma SMMC7721 cells, and human colorectal carcinoma SW620 cells were purchased from American Type Culture Collection (ATCC, Manassas, VA, USA). All cell lines were maintained in Dulbecco’s Modified Eagle Medium (DMEM) media supplemented with fetal bovine serum (10%), penicillin (100 units/mL), and streptomycin (50 units/mL) at 37 °C in a CO_2_ incubator (95% relative humidity, 5% CO_2_).

#### 2.4.2. MTT Assay

Cell viability was confirmed by measuring the ability of cells to transform 3-(4,5-dimethylthia-zol-2-yl)-2,5-diphenyltetrazolium bromide (MTT) to a purple formazan dye [37]. Cells were seeded in 96-well tissue culture plates (3 × 10^3^ cells per well) for 24 h. The cells were then incubated with the tested compounds at different concentrations for different periods of time. After incubation, 20 μL per well of MTT solution (5 mg/mL in phosphate buffered saline, PBS) was added, followed by incubation for a further 5 h. The medium was aspirated and replaced with 150 μL/well of DMSO to dissolve the formazan salt formed. The colour intensity, which reflects the cell growth condition, was measured at 570 nm using a microplate spectrophotometer (SpectroAmaxTM250, BioTek Instruments, Inc., Winooski, VT, USA).

#### 2.4.3. Flow Cytometric Analysis

The apoptosis rate was analysed by flow cytometry, as previously described [38]. Treated or untreated cells were trypsinised, washed with PBS, and then fixed in 75% ethanol overnight at −20 °C. Next, the fixed cells were washed with PBS and stained with propidium iodide (PI) for 4 h in the dark. Finally, the above-described cells were analyzed with an Epics XL-MCL flow cytometer (Beckman Coulter, Miami, FL, USA).

#### 2.4.4. Bcl-2 G-Quadruplex DNA-Binding Studies

##### Electronic Spectra

Electronic spectra were recorded to clarify the interaction of complexes **1b** with CT-DNA and bcl-2 G-quadruplex DNA. The absorption titration of the Ru(II) complex in Tris-HCl buffer was performed by using a fixed complex concentration to which increments of the DNA stock solution were added. The concentration of the **1b** solution was 20 μM. Next, we performed two experiments in which one group of CT-DNA was added by degrees. The other group of bcl-2 G-quadruplex DNA was added by degrees. Complex-DNA solutions were allowed to incubate for 3 min before the absorption spectra were recorded [39]. The titration processes were repeated several times until no change was observed in the spectra indicating the binding saturation has been achieved. The changes in the Ru(II) complex concentration caused by dilution at the end of each titration were negligible. The intrinsic binding constant (K) for **1b** with DNA at IL absorption were calculated following Equations (1) and (2), according to the decay of IL absorption in the presence of DNA [39]. The intrinsic binding constant *Kb* of arene Ru(II) complex to DNA was calculated from the following Equation

(ε_a_ − ε_f_)/(ε_b_ − ε_f_) = [*b* − (b^2^ − 2*K*^2^C_t_[DNA]/*S*)]^1/2^/2KC_t_ (1)

*B* = 1 + *K*C_t_ + *K*[DNA]/2S (2)

[DNA] is the concentration of DNA. The apparent absorption coefficients ε_a_ corresponds to the extinction coefficient observed (A_obsd_/[M]). ε_f_ corresponds to the extinction coefficient of the free compound. ε_b_ is the extinction coefficient of the compound when fully bound to DNA, and *K* is the intrinsic binding constant in M^−1^. C_t_ is the total metal complex concentration, and *S* is the binding size. The ratio of slope to intercept in the plot of (ε_a_ − ε_f_)/(ε_b_ − ε_f_) *versus* [DNA] gave the value of K.

##### EB-Quenching Experiments

Fluorescence spectroscopy measurements were performed on a RF-5301 fluorescence spectrophotometer using a 1 cm path length quartz cell. Fluorescence quenching of EB-DNA system can be used for a compound having an affinity to DNA in spite of its binding mode. This method only measures the ability of the compound to affect the EB fluorescence intensities in the EB-DNA system [40]. The fluorescence spectra of EB were measured using an excitation wavelength of 520 nm, and the emission range was set between 550 and 750 nm. The spectra were analyzed according to the classical Stern-Volmer equation [41]:

*I*_0_/*I* = 1 + *Ksv* [*Q*] (3)

where *I*_0_ and *I* are the fluorescence intensities at 599 nm in the absence and presence of the quencher, respectively, Ksv is the linear Stern–Volmer quenching constant, and [*Q*] is the concentration of the quencher. In these experiments, [EB] = 16 μM, [CT DNA] = 1 mM, [*bcl-2* G4 DNA] = 100 μM.

##### Circular Dichroism

To gain further information, we also recorded CD spectra of DNA that was modified by **1b**. CD spectral characteristics were compared for CT-DNA and bcl-2 G-quadruplex DNA as a function of increasing concentrations of **1b**. Moreover, **1b** has no intrinsic CD signals, as they are achiral, so that any CD signal above 300 nm can be attributed to the interaction of the complex with DNA [42].

##### CD Melting

Typical CD melting curves were obtained by following changes in ellipticity as a function of temperature at 262 or 292 nm. The mixture solutions consisting of 10 μM of complex **1b** and 100 μM of CT-DNA, and the solutions of 100 μM CT-DNA were respectively heated at a rate of 0.1 °C/min in quartz cuvettes with a path length of 0.1 or 1.0 cm, which resulted in the collection of two data points/°C. The mixture solutions of 10 μmol complex **1b** and 100 μM *bcl-2* G-quadruplex DNA, and the solutions of 100 μM CT-DNA, were respectively processed by the same method. A Circular Dichroism Spectrometer was used to measure the CD signal of the four solutions defined above at 280 nm with changes in temperature [43].

## 3. Results and Discussion

### 3.1. Microwave-Assisted Synthesis of Arene Ru(II) Complexes

**Figure 1:**
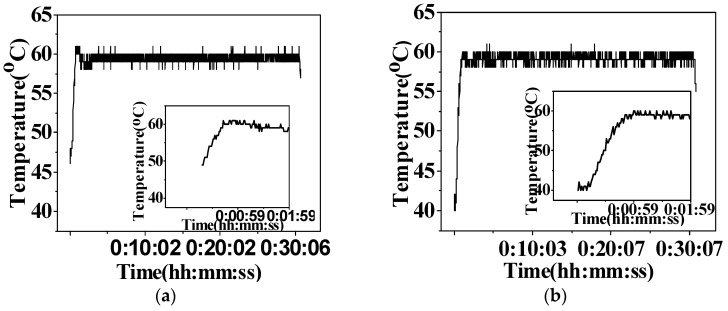

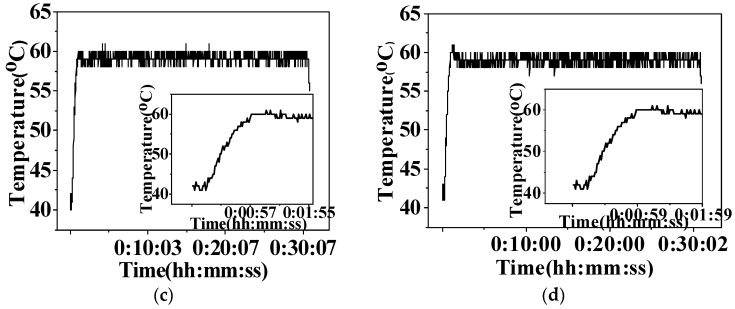
Reaction profile of arene Ru(II) complexes in dichloromethane irradiated by microwave for 30 min at 60 °C. (**a**) **1b**; (**b**) **2b**; (**c**) **3b**; (**d**) **4b**.

Microwave-assisted synthesis is emerging as a rapidly-expanding method in organic and inorganic synthesis chemistry. The application of microwave irradiation [44] has significantly increased the yield for most complexes to about 90%, which exceeded that of conventional synthesis methods (Table 1). We can appreciate that the microwave heating process in more detail from a reaction profile (*i.e.*, the heating curves of temperature *versus* time (hh:mm:ss)). As shown in Figure 1, under irradiation conditions, the temperature of the reaction mixture reached 60 °C in about 60 s, and sustained a stable temperature during the whole reaction process. The microwave-assisted synthesis method has the advantage of improving the stability of the reaction progress to prepare the arene Ru(II) complexes. The yields of the targeted complexes was approximately 90%, which exceeded that of conventional heating methods.

### 3.2. Inhibit the Growth of A549 Cells through Inducing Cell Apoptosis

The *in vitro* anti-cancer activities of the arene Ru(II) complexes were screened against the human lung adenocarcinoma A549 cells, human hepatocarcinoma SMCC7721 cells, and human colorectal carcinoma SW620 cells by the MTT assay after a 72 h treatment, in which, *cis*-platin was used as a positive control (Table 2). The results indicated that **1b** was not only the most promising growth inhibitor against A549 cells, but also exhibited an anti-tumor activity (IC_50_) of 16.59 μmol, which was at similar levels to cis-platin (IC_50_ = 16.54 μmol). In addition, arene Ru(II) complexes with the same substituents in different positions, such as –Cl residue in a para-position (**1b**), exhibited higher anti-tumor activities against A549 cells than that seen with complexes with substituents in a meta-position (**2b**). Arene Ru(II) complexes with –Cl substitutions in a para-position (**1b**) exhibited higher anti-tumor activity against A549 cells than that of complexes with –NO_2_ substituents in a para-position (**3b**), an observation that indicated the electron donating ability of –Cl was more potent than –NO_2_.

**Figure 2:**
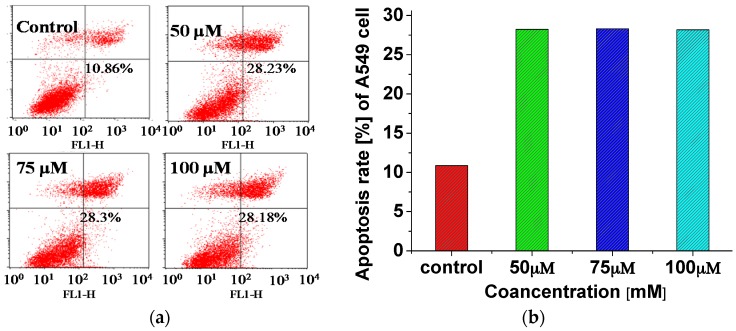
(**a**) Flow cytometric (FCM) analysis of the apoptosis of A549 cells treated with complex **1b** after 24 h. The effect of **1b** (50, 75, and 100 μM) on A549 cell apoptosis was determined by flow cytometry. Untreated (control) cells or cells treated for 24 h were harvested, fixed, stained with Annexin V/PI, and assessed for cell apoptosis distribution by flow cytometric analysis; (**b**) Percentages of apoptotic cells were measured by the annexin V/PI flow cytometry analysis, as described in the Experimental section. The y-axis plots the sum of early and late apoptotic cells as the mean ± standard deviation of three independent experiments.

To examine whether arene Ru(II) complex-induced growth inhibition was the result of apoptosis, flow cytometric analysis was employed to investigate the underlying mechanisms regulating cell death that might have been formally induced by one of the arene Ru(II) complexes, **1b**. Furthermore, A549 cells were exposed to different concentrations of **1b** (0, 50, 75, and 100 μM) for 24 h and examined by flow cytometric (FCM) analysis. Results showed a prominent increase in cell apoptosis at 24 h (Figure 2). Upon increasing the concentration of **1b**, the level of apoptosis increased markedly, and reached a value of 28.2% for the 5 μM experiment, which was approximately three-fold higher than that of the control (10.86%), which also indicated that the arene Ru(II) complex might have inhibited the growth of A549 cells by induction of apoptosis.

### 3.3. Bindingand Stabilizing bcl-2 G-Quadruplex DNA

The Bcl-2 protein, which is the product of the *bcl-2* proto-oncogene, is a mitochondrial membrane protein, which exists in delicate balance with other related proteins and takes part in controlling apoptosis or programmed cell death. Over-expression of the *bcl-2* proto-oncogene in tumor cells causes resistance to chemotherapy or radiotherapy-induced apoptosis [27]. Due to the apoptosis-related functions of Bcl-2 and the regulatory role of the G-quadruplex on bcl-2 [29], the G-quadruplex ligands may become potential anti-tumor agents. Thus, the DNA-binding properties of complex **1b** with the *bcl-2* G-quadruplex DNA was studied next with various techniques.

#### 3.3.1. Electronic Spectra

Electronic absorption spectroscopy is one of the most common methods used to study the interaction of ruthenium complexes with DNA. In general, ruthenium complexes have characteristic spectroscopic properties, which undergo hypochromism and red shift in the presence of DNA. The degree of the observed change is usually proportional to the binding strength.

**Figure 3:**
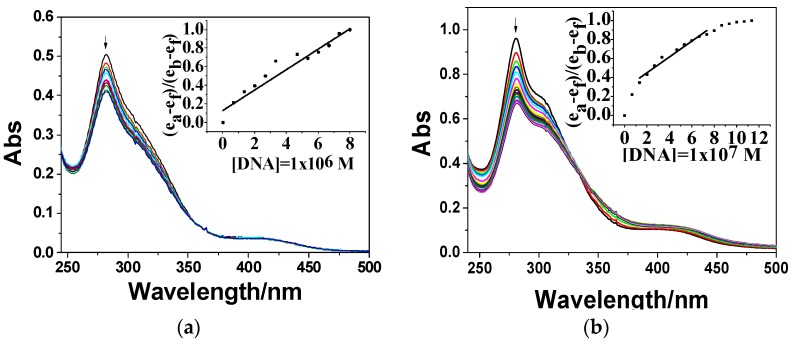
The electronic absorption spectra of complex **1b** interacted with (**a**) calf thymus DNA (CT-DNA); and (**b**) *bcl-2* G-quadruplex DNA. [Ru] = 20 μmol, [CT-DNA] = 1 μM, [*bcl-2* G4-DNA] = 100 μM. The arrow shows the emission intensity changes upon increasing DNA concentration.

As shown in Figure 3, upon the addition of CT-DNA or *bcl-2* G-quadruplex DNA, clear evidence of hypochromism (18.6%, 30.4%) at LMCT (ligand-to-metal charge transition) absorption at 282 and 281 nm, were respectively observed in the spectra of **1b**, with the intrinsic binding constant (K) calculated according to the decay of LMCT absorption to approximately 8.59 × 10^5^ M^−1^ and 2.94 × 10^6^ M^−1^, respectively. These data pointed to a certain interaction between **1b** and CT-DNA, *bcl-2* G-quadruplex DNA. Furthermore, **1b** showed a stronger binding ability with *bcl-2* G-quadruplex DNA. These interactions were further confirmed by the following fluorescence quenching experiment.

#### 3.3.2. Fluorescence Quenching

Due to the low fluorescence of **1b** in Tris-HCl buffer (pH = 7.2), fluorescence quenching of ethidium bromide (EB) and DNA was carried out to further study the DNA-binding behaviour of **1b**. Fluorescence quenching can be used to study the affinity of a complex to DNA, in spite of its binding mode [42].

**Figure 4:**
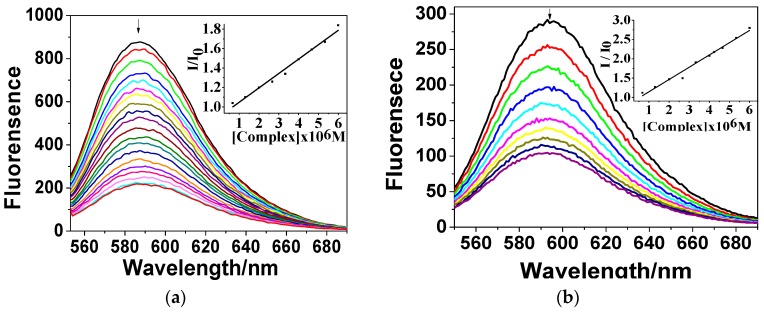
Emission spectra of ethidium bromide DNA (EB-DNA) in the incubation buffer in the absence and presence of complex **1b**. [EB] = 16 μM, [CT DNA] = 1 μM, [*bcl-2* G4 DNA] = 100 μM. Arrow shows the emission intensity changes upon increasing concentration of **1b**. (**a**) EB-CT-DNA; (**b**) EB-*bcl-2* G-quadruplex DNA.

Upon increasing the concentration of complex **1b**, the emission intensity of the EB-DNA system decreased dramatically (in Figure 4), implying the possibility that **1b** provided a competitive combination with CT-DNA and *bcl-2* G-quadruplex DNA by displacing EB [45,46]. Stern–Volmer plots of EB-DNA (Figure 4) illustrate that the quenching of EB bound to DNA by the compounds with the linear Stern–Volmer equation, which proves that the displacement of EB bound to DNA by each compound results in a decrease in the fluorescence intensity. The Ksv values of the complex, which is the Stern–Volmer quenching constant, show that they can displace EB and bind to the DNA [47]. The Ksv values for CT-DNA and *bcl-2* G4-DNA are 1.48 × 10^5^  M^−1^ and 3.18 × 10^5^  M^−1^, respectively. The data suggest the interaction. Moreover, the binding strength of **1b** with *bcl-2* G4-DNA is greater than with CT-DNA.

#### 3.3.3. Circular Dichroism

**Figure 5:**
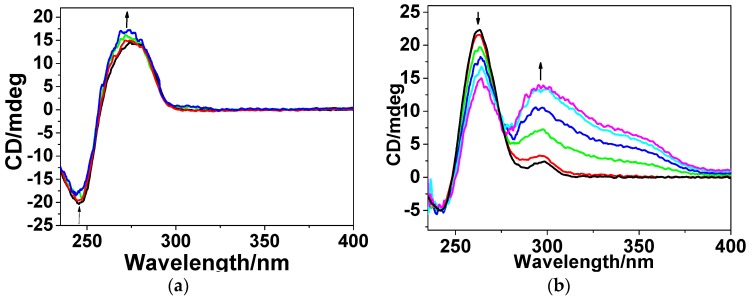
The change of circular dichroism (CD) spectra of (**a**) CT-DNA; and (**b**) *bcl-2* G-quadruplex DNA with the increasing concentration of complex **1b**. [DNA] = 100 μM, [Ru] = 0, 2, 4 μM.

CD titration experiments were also carried out to investigate the conformational change of CT-DNA and *bcl-2* G-quadruplex DNA in the presence of arene Ru(II) complexes, and the results are shown in Figure 5. The CD spectra of CT-DNA exhibited a strong positive signal in the range of 250~300 nm with the maximum at 280 nm. When **1b** was added to the solutions, the positive CD signal of CT-DNA increased distinctly. The strength of the positive CD signal at 263 nm increased by 16.3%, which indicated that **1b** affected the CT-DNA conformation.

In Figure 5b, the decrease in the strength of the positive CD signal of *bcl-2* DNA at 263 nm was 33.9%. Besides, there was also a large induced CD signal in the range of 290–400 nm. It is noteworthy that there was a distinctive positively-induced CD signal observed in the range of 300–400 nm, which has been considered as proof that the complex may bind to the *bcl-2* G-quadruplex DNA in the groove binding mode [9,48,49,50].

These results indicated that arene Ru(II) complexes **1b** exercise a great influence both on CT-DNA and *bcl-2* G-quadruplex DNA conformations. It can stabilize and bind to the structure of the *bcl-2* G-quadruplex DNA in groove binding mode, with stronger binding abilities that exceeded those seen more than to CT-DNA.

#### 3.3.4. Melting Point Experiments

Melting point (Tm) studies were conducted to further examine the conformation of DNA after binding **1b**. In general, double-stranded DNA will gradually dissociate to single strands following an increase in temperature, which can be observed by examining the decrease in the CD at 280 nm [44]. A large change in the Tm of DNA indicates a strong interaction between a complex and DNA.

**Figure 6:**
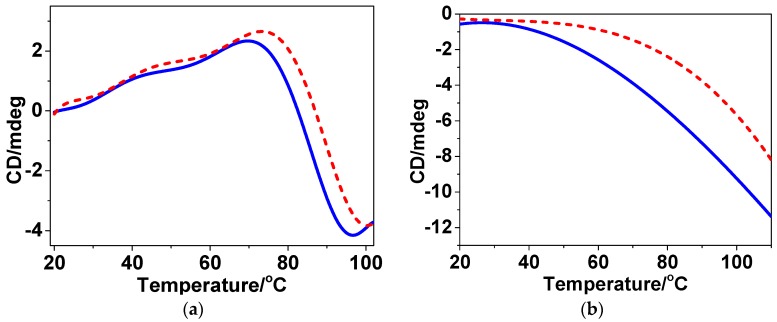
Typical CD melting curves of (**a**) CT-DNA; and (**b**) *bcl-2* G-quadruplex DNA without (—) or with (- - -) **1b** in the incubation buffer. The CD signal was recorded with a positive band at 280 nm. [DNA] = 100 μM; [Ru] = 10 μM.

The results shown in Figure 6 suggest that **1b** may bind to CT-DNA and *bcl-2* G-quadruplex DNA, which is evidenced by a noticeable rise in the melting point of CT-DNA and *bcl-2* G-quadruplex DNA following exposure to **1b**. By contrast, the ΔTm values of the CT-DNA and *bcl-2* G-quadruplex DNA with **1b** were respectively 1.6 and 4.2 °C. Taken together, these results clearly indicate that the Ru(II) complex **1b** displays a stronger stabilizing ability on the conformation of *bcl-2* G-quadruplex DNA.

## 4. Conclusions

In summary, four arene RuII complexes were synthesized in a time period of 30 min using microwave-assisted technology, under conditions of rapid rising to the required reaction temperature (<60 s) and no obvious fluctuation of temperature in the reaction process. According to results obtained from the MTT assays, **1b** exhibited selective inhibition of A549 cells at similar levels that were obtained with cisplatin. Furthermore, studies by spectroscopy and melting point experiments demonstrated that arene Ru(II) complexes exhibited excellent binding affinity to *bcl-2* G-quadruplex DNA in the groove-binding mode, and displayed potential application as an apoptotic inducer of A549 cells through a mechanism that stabilizes *bcl-2* G-quadruplex DNA. Taken together, our results suggest that synthetic complexes could formally target G-quadruplex DNA and stabilize its structure in such a way to induce apoptosis of tumor cells. This approach could serve as a class of dual functional anti-tumor agent therapy with potential application in cancer chemoprevention and chemotherapy.

## Figures and Tables

**Table 1:** Yields of arene Ru(II) complexes by microwave-assisted and conventional synthesis methods.

Complexes	Microwave-Assisted			Conventional		
	Temperature/°C	Time/h	Yield/%	Temperature/°C	Time/h	Yield/%
**1b**	60	0.5	90.7	60	4	76.2
**2b**	60	0.5	92.7	60	4	81.2
**3b**	60	0.5	89.9	60	4	70.6
**4b**	60	0.5	91.7	60	4	76.0

**Table 2:** The anti-tumor activity of arene Ru(II) complex against various tumor cell lines after 72 h of incubation, determined using the MTT assay.

Complexes	IC_50_/μmol		
	A549	SMCC7721	SW620
**1b**	16.59	>100	>100
**2b**	26.55	>100	>100
**3b**	>100	>100	>100
**4b**	91.88	>100	>100
[Ru(arene)Cl_2_]_2_Cl_2_	73.19	>100	>100
*Cis*-platin	16.54	2.92	5.74

## Supplementary Materials

The following are available online at www.mdpi.com/1996-1944/9/5/386/s1. Figure S1: The ESI-MS spectra of complex **a**: **1b**, **b**: **2b**, **c**: **3b**, **d**: **4b**; Figure S2: The ^1^H NMR and ^13^C NMR spectra of complex **1b**; Figure S3: The ^1^H NMR and ^13^C NMR spectra of complex **2b**; Figure S4: The ^1^H NMR and ^13^C NMR spectra of complex **3b**; Figure S5: The ^1^H NMR and ^13^C NMR spectra of complex **4b**.
