# Supplementary material for: Microwave-Assisted Synthesis of Arene Ru(II) Complexes Induce Tumor Cell Apoptosis Through Selectively Binding and Stabilizing bcl-2 G-Quadruplex DNA
Source: Materials (Basel). 2016 May 17;9(5):386. doi: 10.3390/ma9050386 (PMC5503023; doi:10.3390/ma9050386)
Supplement: Supplementary file 1 [file materials-09-00386-s001.pdf]

Supplementary Materials: Microwave-Assisted Synthesis of Arene Ru(II) Complexes Induce Tumor Cell Apoptosis Through Selectively Binding and Stabilizing *bcl-2* G-Quadruplex DNA

Yanhua Chen, Qiong Wu, Xicheng Wang, Qiang Xie, Yunyun Tang, Yutao Lan, Shuangyan Zhang and Wenjie Mei

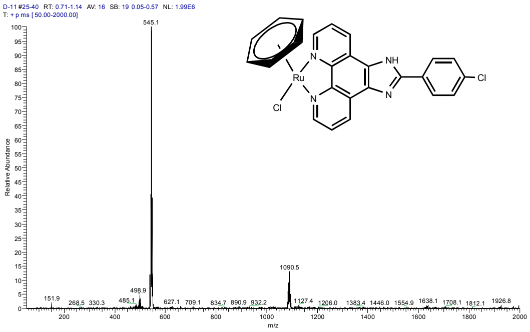

(**1b**)

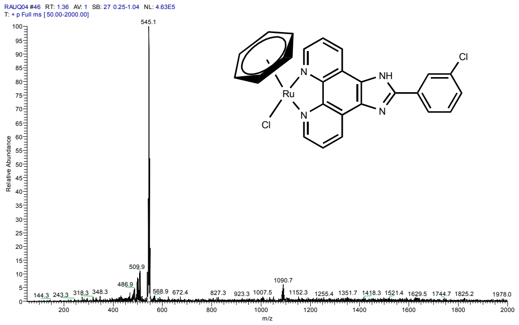

(**2b**)

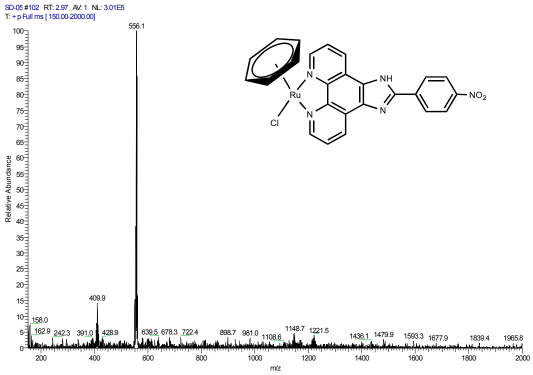

(**3b**)

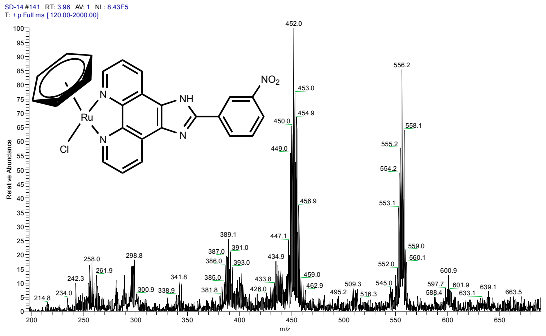

(**4b**)

Figure S1. The ESI-MS spectra of complex **1b**, **2b**, **3b**, **4b**.

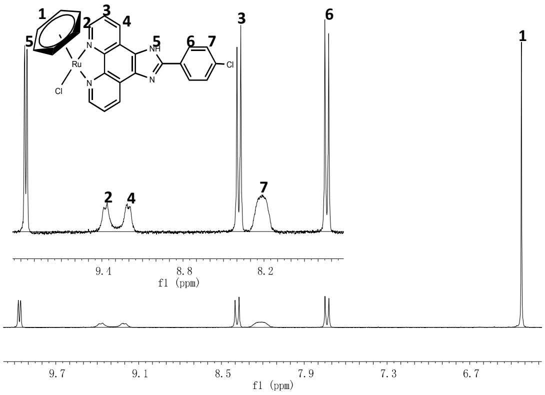

(**a**)

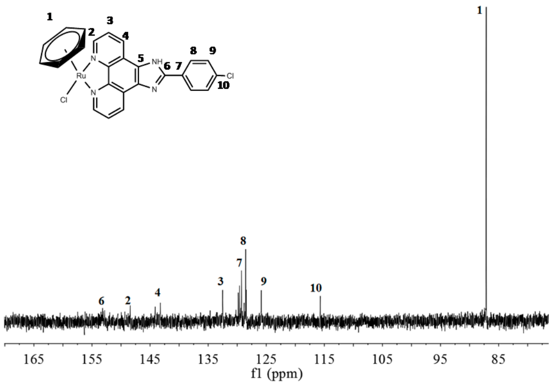

(**b**)

Figure S2. The ^{1}H NMR (**a**) and ^{13}C NMR (**b**) spectra of complex **1b**.

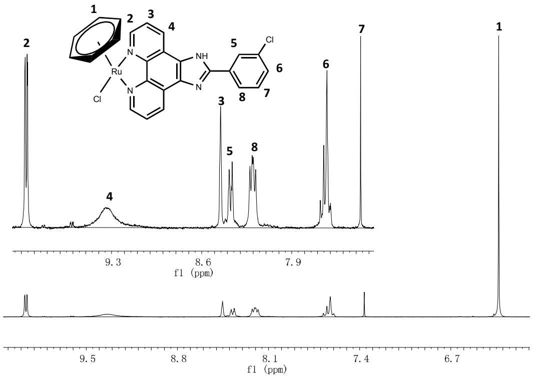

(**a**)

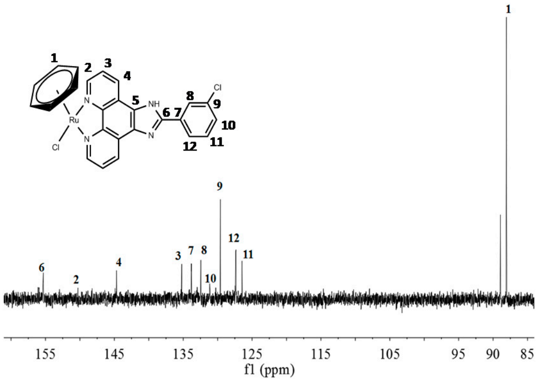

(**b**)

Figure S3. The ^{1}H NMR (**a**) and ^{13}C NMR (**b**) spectra of complex **2b**.

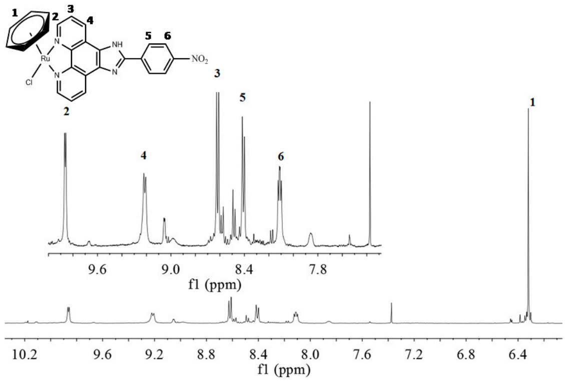

(**a**)

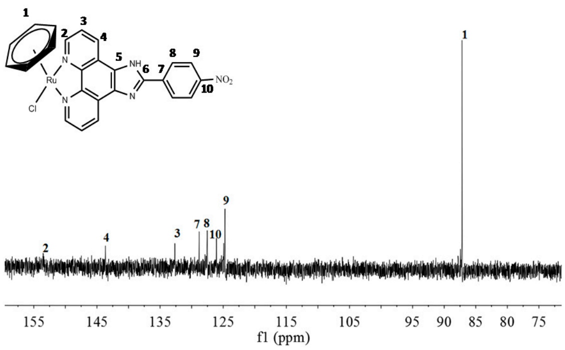

(**b**)

Figure S4. The ^{1}H NMR (**a**) and ^{13}C NMR (**b**) spectra of complex **3b**.

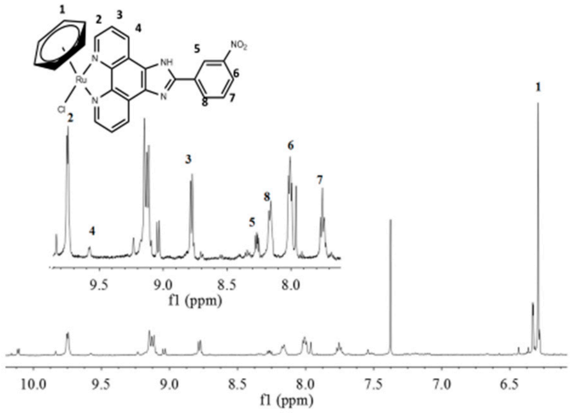

(**a**)

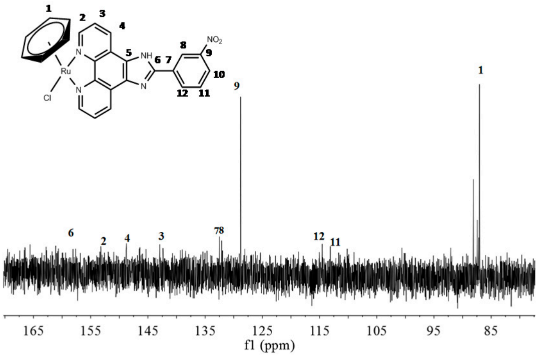

(**b**)

Figure S5. The ^{1}H NMR (**a**) and ^{13}C NMR (**b**) spectra of complex **4b**.
